# Erector Spinae Plane Catheter for Postoperative Thoracotomy Pain in a Patient With Indwelling Spinal Cord Stimulators: A Case Report

**DOI:** 10.7759/cureus.30069

**Published:** 2022-10-08

**Authors:** David T Cheng, Eldhose Abrahams, Aimee Pak

**Affiliations:** 1 Anesthesiology, University of Oklahoma Health Sciences Center, Oklahoma City, USA

**Keywords:** case report, post-thoracotomy pain syndrome, thoracotomy pain, nerve block, regional anesthesia, postoperative pain, spinal cord stimulator, erector spinae plane block

## Abstract

Analgesia after thoracotomy is challenging but important as inadequate pain control may result in early postoperative complications and a higher risk for post-thoracotomy pain syndrome. The authors report the successful utilization of an erector spinae plane (ESP) catheter for post-thoracotomy analgesia in a 40-year-old female with two dual-leaded spinal cord stimulators (SCS) in the cervical and thoracic levels. Although thoracic epidural analgesia is the current standard, epidural catheterization may present with obstructive, mechanical, or infectious concerns in patients with SCS. The ESP block may be a preferable approach to postoperative analgesia after thoraco-abdominal surgery over the thoracic epidural for patients with SCS.

## Introduction

Pain associated with thoracotomy is widely known for its intensity as well as its persistence long after the operation is completed [1]. Adequate pain control significantly improves forced vital capacity and forced expiratory volume in one second when comparing thoracic epidural analgesia against intravenous analgesia after thoracic surgery [2]. This consequently reduces the risk of developing pneumonia by nearly 50%, as well as other postoperative pulmonary complications such as prolonged ventilation beyond 24 hours and reintubation [3]. Furthermore, adequate analgesia decreases cardiac complications, such as myocardial infarction and supraventricular tachyarrhythmias, after thoracic surgery [1,3]. Post-thoracotomy pain has been shown to be modifiable, with a decrease in chronic pain sequelae from 50-78% to 21-45% when perioperative pain is sufficiently managed [4,5]. In fact, the intensity of acute postoperative thoracotomy pain directly predicts the development of chronic post-thoracotomy pain syndrome, which is defined as pain along the thoracotomy site persisting for greater than two months after surgery [6]. Patients suffering from this syndrome also experience pain-related disabilities in other aspects of life such as relationships, sleep, mood, ability to work, and enjoyment of life [7].

The current gold standard for post-thoracotomy pain is thoracic epidural analgesia [8]; however, there is a dearth of literature on neuraxial anesthesia in patients with spinal cord stimulators (SCS). The European Society of Regional Anaesthesia recommends a paravertebral block or thoracic epidural analgesia as the first option, otherwise intravenous patient-controlled analgesia (PCA) with a strong opioid if those techniques are contraindicated [9].

For our patient, the presence of indwelling SCS precluded a thoracic epidural or paravertebral block as viable options due to the risk of mechanical damage to SCS leads. Bacteremia and infection seeding to the device, as well as patchy or incomplete analgesia, are other significant risks. In addition, we chose to avoid opioid monotherapy for postoperative analgesia to mitigate the numerous dose-dependent adverse effect of opioids including but not limited to respiratory depression, sedation, ileus, and risk for prolonged opioid use after surgery [10]. With these considerations, we elected to perform an erector spinae plane (ESP) block since it is outside of the epidural space and far from the two SCS leads. To our knowledge, this is the first case of ESP catheter placement for postoperative analgesia due to the presence of an indwelling SCS. Written Health Insurance and Portability Act (HIPAA) authorization was obtained from the patient for the publication of this case presentation. This manuscript adheres to the CAse REports (CARE) guidelines.

## Case presentation

A 40-year-old wheelchair-bound American Society of Anesthesiologists (ASA) physical status III patient with a history of cerebral palsy, esophageal spasms, cervical and thoracic SCS devices for chronic chest and epigastric pain, presented for surgical repair of recurrent paraesophageal hernia, fundoplication takedown, and partial gastrectomy via a left thoracotomy approach. She presented with moderate extremity contractures and was able to verbalize and speak appropriately though with apparent developmental delay. She quantified her baseline pain as 4/10 using the Numeric Rating Scale. Our Acute Pain Team was consulted for assistance in postoperative pain management. According to the patient and her mother, the SCS devices were deactivated for many years and were not being actively managed by her current pain physician. Re-activation or further outpatient follow-up was neither desired nor pursued at the time. Her daily pain medications included duloxetine 20 milligrams, gabapentin 100 milligrams, amitriptyline 100 milligrams, and naproxen as needed.

There were no available records of either SCS device, but both were visualized on a preoperative chest radiograph upon chart review. The cervical SCS leads were midline, extending to the C2 vertebral body proximally and tunneled to a right-sided generator. The thoracic SCS leads were midline and located at the superior endplate of the T7 vertebral body with its distal lead tunneled inferiorly towards a left-sided generator. An upper thoracic epidural placement would be needed for post-surgical analgesia; however, due to the proximity of the two SCS and respective paired leads within the cervical and thoracic spaces, a left-sided ESP block and catheter was offered instead of a thoracic epidural catheter. After a discussion of risks and benefits with the patient and her mother, informed consent was obtained.

**Figure 1:**
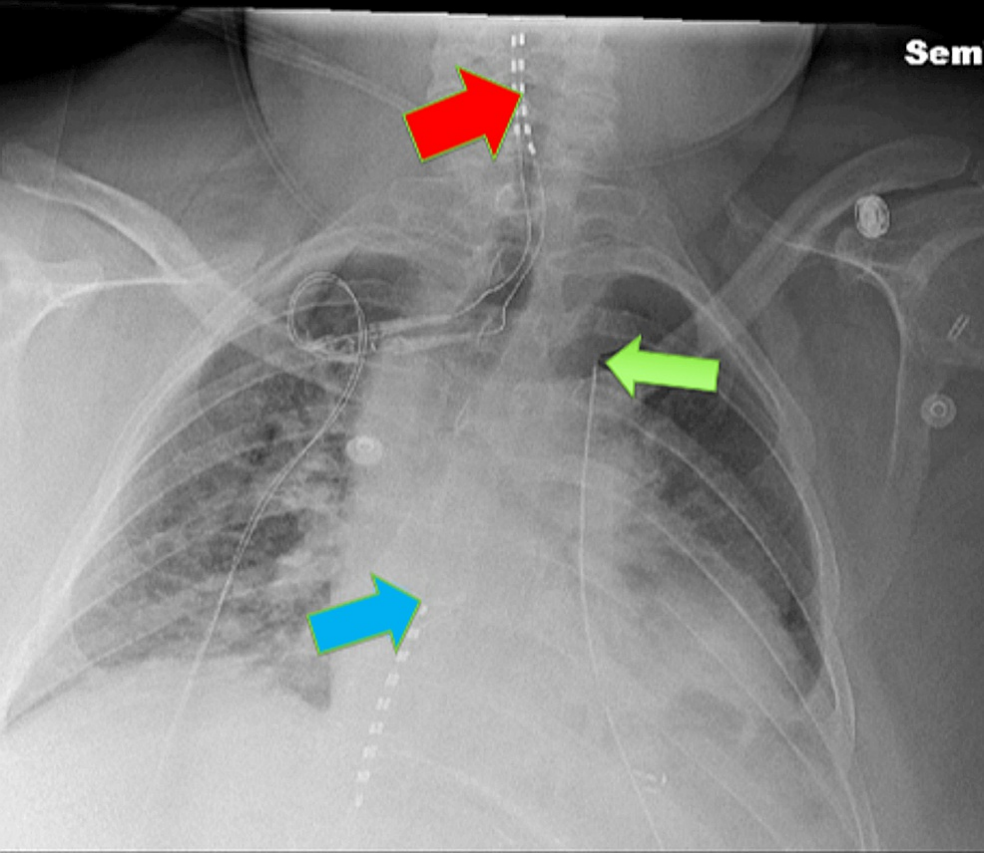
Postoperative chest radiograph A postoperative chest radiograph was obtained on postoperative day one. The cervical spinal cord stimulator has two cylindrical leads (red arrow) which can be visualized extending to the C2 vertebral body proximally and tunneled inferiorly to a presumed right-sided generator (not visible). The thoracic spinal cord stimulator has two cylindrical leads (blue arrow) which can be visualized at the superior endplate of the T7 vertebral body with its distal lead tunneled inferiorly towards to left-sided generator (not fully visible). The erector spinae plane catheter (light green arrow) overlies the left T3 to T6 transverse processes. The catheter is directed cranially with its tip terminating between the T3 and T4 transverse processes.

After induction of general anesthesia, the patient was placed in the right lateral decubitus position. Her back was cleansed with 2% chlorhexidine skin prep and sterile draping was placed. A 15-4 MHz linear ultrasound transducer (Sonosite SII, Fujifilm, Bothell, Washington, United States) was placed in the sagittal orientation at the most lateral aspect of the T6 transverse process on the left side. The erector spinae muscle complex was identified superficial to the transverse process. The 18-gauge Tuohy needle (Contiplex®, B. Braun Medical Inc., Melsungen, Germany) was advanced in-plane in a cephalad direction and injection of 20 milliliters of 0.25% bupivacaine was accomplished easily with dissection of the erector spinae muscle from the transverse process. A 20-gauge polyamide nylon closed-tip catheter (Perifix®, B. Braun Medical Inc., Melsungen, Germany) was then inserted with its tip visualized under ultrasound between the T3-T4 transverse processes and the catheter was secured. The patient remained hemodynamically stable under general anesthesia throughout the procedure, and a postoperative chest X-ray was reviewed (Figure 1).

Additionally, a multimodal pain regimen was initiated after surgery, which included continuing her home gabapentin and duloxetine as well as scheduled acetaminophen, scheduled methocarbamol, and hydromorphone PCA. The ESP catheter was maintained at a continuous infusion of 0.25% bupivacaine at 6 milliliters per hour.

On postoperative day (POD) one, the patient self-described her pain as “great” since she was mostly experiencing her baseline (4/10) pain at rest, though it did increase to 6/10 with deep inspiration and cough. She used 48 morphine milligram equivalents (MME) from her hydromorphone PCA. Her mood was good, and she did not complain of issues with sleep overnight. On POD two, she started physical therapy so that she could transfer into her motorized wheelchair, which caused higher pain scores and opioid use. She reported 6/10 pain at rest and 8/10 with activity, with 68 MME use. She required a 10 milliliter 0.375% bupivacaine bolus twice over 12 hours apart. While sensory testing was precluded by surgical dressings and her contractures, the significant relief reported solely after bolus administration and correlating improvement in vital signs strongly suggested catheter efficacy. This allowed her to sleep well overnight. On POD three, her pain was 4/10 at rest and 7/10 with activity and participation in physical therapy, and without opioid utilization from the available PCA. Her chest tube was removed on this day, followed by the removal of the ESP catheter. She continued to sleep comfortably overnight. Her pain was managed with non-opioid analgesics thereafter (scheduled acetaminophen and methocarbamol) and she was discharged to home on POD six. On subsequent follow-up with her cardiothoracic surgeon after discharge, the patient did complain of any thoracotomy pain.

## Discussion

SCS have gained wider application in patients with chronic neuropathic pain conditions such as failed back surgery syndrome, complex regional pain syndrome, peripheral neuropathy, refractory angina pectoris, and phantom limb pain [11]. There is a general trend towards implantation in younger patient populations (aged 45 to 64 years), particularly in females, according to demographic studies [12]. The implication is that there is a higher likelihood to encounter SCS patients in the perioperative, traumatic, or obstetric settings and evaluation for interventional analgesia.

There are several considerations to epidural placement in a patient with SCS. Bull et al. advocate absolute contraindication of neuraxial procedures in such cases because they can cause direct damage to SCS leads as well as possibly introduce infection [13]. Harned et al. counter that the risk of device damage can be mitigated simply with prior knowledge of its implanted location and avoidance of needle entry at the stimulator or lead levels [14]. Neuraxial analgesia may be carefully performed below the level of the SCS and lead entry, preferably with fluoroscopic guidance [12], and with strict attention to aseptic technique [13,14]. Despite concerns of transient bacteremia with neuraxial procedures, routine antibiotic prophylaxis is not advised [13-15].

Also, while unlikely for epidurally-administered medications to cause indirect damage due to the protective fibrosis that is known to develop around the leads, that same fibrosis may compromise epidural spread and lead to patchy or incomplete coverage [13,14]. In the field of obstetric anesthesia, there have been several case reports of lumbar epidural placement in patients with cervical or thoracic SCS with variable success. One case series reported a 25% epidural failure rate in four SCS parturients who received labor epidural analgesia, which contrasts with the overall failure rate of 6.8% in the general population [15]. In all cases, labor epidural catheters were placed in the lumbar interspaces. The literature to date contains mixed opinions about lumbar epidural placement on parturients with SCS.

At the time of this manuscript, the authors could not find any reports on thoracic epidural placement in patients with SCS. This may reflect hesitancy with epidural placement, usually inserted at the T6 to T10 levels for incisional coverage, since SCS placement for low back and leg pain would coincide with those levels as well [14]. Published expert opinion, though few, are against thoracic epidural placement with an indwelling SCS [13,14]. With such limitations, we propose that the ESP block and catheter placement should be indicated for postoperative analgesia after thoracolumbar surgeries in patients with indwelling neuraxial devices.

The ESP block is an interfascial plane injection between the transverse process and the erector spinae muscle [16]. At the thoracic level, local anesthetic is proposed to anesthetize the exiting spinal nerves with potential spread into the paravertebral and epidural spaces, as well as laterally to the intercostal space based on cadaveric and radiologic studies [17]. The injection site is superficial and lateral to the epidural space, which is far from an existing SCS device and leads.

Several studies have demonstrated the utility of ESP blocks in the treatment of pain from thoracic surgery and reduced opioid use. In a 2020 randomized, blinded clinical trial of 60 patients undergoing thoracotomy, there was decreased morphine use and lower pain scores in subjects who received an ESP single-shot block plus conventional opioid analgesia for postoperative pain compared to subjects who only used conventional opioid analgesia [18]. In a case-control study in 2021, Zengin et al.* *demonstrated efficacy in a combined erector spinae plane block plus thoracic paravertebral block for patients undergoing video-assisted thoracic surgery [19]. This study showed effective postoperative pain control with decreased opioid requirements in patients in which thoracic epidural analgesia may be contraindicated. While the presence of pre-existing chronic pain in our patient makes a comparison of pain levels to existing literature challenging, it should be noted that our patient reported a two-point change in pain score at rest (4/10, same as baseline) and with inspiration or cough (6/10) in POD one, which is a similar differential at 24-hours postoperatively to mean reported levels in patients without chronic pain who received thoracic epidural analgesia at the end of surgery [5].

In addition to analgesia for acute surgical pain, the ESP block is a viable treatment for post-thoracotomy pain syndrome (PTPS), which is surgical pain that persists more than two months after surgery. In one case series, five patients with PTPS approximately three to seven months after surgery were successfully treated with a single injection ESP block [20].

There is a paucity of studies on ESP blocks performed on patients with SCS for postoperative analgesia after thoraco-abdominal surgeries. We found only one published report of successful ESP block utilization for a patient with a cervical SCS; however, the block was to provide analgesia from the acute pain caused by the tunneling pathway created in the subcutaneous layer to connect the newly placed SCS to its implantable pulse generator [21].

## Conclusions

With expanding application of SCS to reduce opioid reliance in patients with chronic pain, there is a higher likelihood of evaluating these patients for regional analgesic options in the operative, traumatic, or obstetric setting. Thoracic epidural catheterization may not be an appropriate modality for selected patient populations due to varied risks. Alternatively, the ESP block is efficacious while offering distance from the device regardless of implanted level, technical ease in placement, and the ability for continuous infusion via catheterization. Perhaps with these considerations, ESP block and catheterization should be considered as a regional analgesic option for acute postoperative analgesia after thoracic and abdominal surgeries in patients with indwelling SCS.
